# Heterologous Expression of the Transcription Factor *EsNAC1* in *Arabidopsis* Enhances Abiotic Stress Resistance and Retards Growth by Regulating the Expression of Different Target Genes

**DOI:** 10.3389/fpls.2018.01495

**Published:** 2018-10-15

**Authors:** Can Liu, Qinghua Sun, Lei Zhao, Zhaoxia Li, Zhenghua Peng, Juren Zhang

**Affiliations:** School of Life Sciences, Shandong University, Jinan, China

**Keywords:** abiotic stress tolerance, ChIP-on-chip, NAC transcription factor, plant vegetative development, *Eutrema salsugineum*

## Abstract

Heterologous expression of a transcription factor (TF) gene in a related species is a useful method for crop breeding and the identification of gene function. The differences in phenotype and target gene expression between HE lines (with the heterologous expression of an ortholog) and OX lines (with an overexpressed native gene) must be understood. *EsNAC1*, encoding a NAC protein and the ortholog of *RD26* in *Arabidopsis*, was cloned from *Eutrema salsugineum* and introduced into *Arabidopsis*. The heterologous expression of EsNAC1 retarded the vegetative growth of *Arabidopsis*, and the transgenic plants (HE lines) showed much greater resistance to salt and oxidative stress than the wild type, Col-0. The HE lines accumulated 2.8-fold (8-h light) of starch, 1.42-fold of Chlorophyll a and 1.31-fold of Chlorophyll b than Col-0 during the light period, with obvious differences compared to the RD26OX line. A genome-wide ChIP (chromatin immunoprecipitation analysis)-on-chip assay revealed that EsNAC1 targeted promoters of different genes compared to RD26. In HE lines, EsNAC1 could specifically upregulate the expression level of TF genes *NAC DOMAIN CONTAINING PROTEIN 62 (ANAC062), INTEGRASE-TYPE DNA-BINDING PROTEIN (TINY2)*, and *MYB HYPOCOTYL ELONGATION-RELATED (MYBH)* to show more effective abiotic stress resistance than RD26OX lines. Moreover, *DELTA1-PYRROLINE-5-CARBOXYLATE SYNTHASE 1 (P5CS1), TRYPTOPHAN BIOSYNTHESIS 2 (TRP2)* or *GALACTINOL SYNTHASE 2 (GOLS2)*, was also specifically regulated by EsNAC1 to retard the vegetative growth of HE lines, but not the brassinosteroid singling pathway in RD26OX lines. These differences in phenotypes and metabolism between the HE lines and the RD26OX line implied that the differential features could be produced from the diversity of target genes in the transgenic plants when the ortholog was introduced.

## Introduction

Abiotic stress is a primary factor limiting the quality of agricultural products and yield. Because of the growth of the world population and the global scarcity of water resources, breeding crop varieties that are tolerant of abiotic stress is important. *Eutrema salsugineum* (formerly *Thellungiella salsuginea*) is a valuable model plant for the study of plant stress response mechanisms, particularly salt resistance, because the genome is small and the tolerance to high salinity, drought and low temperatures is high (Zhu, 2001; Inan et al., 2004).

Identification of the genes that play crucial roles in the abiotic stress resistance of plants, such as some transcription factor (TF) genes, has received close attention. Based on their expression characteristics, TFs are divided into two categories, inducible TFs that only express under specific induction conditions, and constitutive TFs that express ubiquitously under normal conditions (Fujita et al., 2004). NAC TFs, one of the largest protein families in higher plants, include more than 107 members in *Arabidopsis* (Riechmann et al., 2000), and some members of this family participate in the regulation of plant development (Guo et al., 2005; Petricka et al., 2012) and/or response to abiotic stress (Tran et al., 2010; Chi et al., 2017). *RESPONSIVE TO DESICCATION 26* (*RD26*, AT4G27410) encodes a member of this family in *Arabidopsis*. RD26 belongs to the ATAF subfamily and is markedly upregulated by salt and drought stress, and the overexpression of RD26 in *Arabidopsis* improves salt stress resistance and retards the growth of plants (Fujita et al., 2004; Tran et al., 2004; Ye et al., 2017). With respect to the results offered by the eFP Browser (Winter et al., 2007), *RD26* shows high expression levels in the mature organs of *Arabidopsis* but is not obviously induced by cold or drought stress (Shiriga et al., 2014). Additionally, *NAC DOMAIN CONTAINING PROTEIN 55* (*ANAC055*) belongs to the same clade of NAC TFs as *RD26* (Hickman et al., 2013) and is involved in dehydration stress response (Ding et al., 2013), abscisic acid (ABA) and jasmonate response (Jiang et al., 2009), and plant senescence (Takasaki et al., 2015). *ANAC016* also plays a role in drought stress (Sakuraba et al., 2015) and oxidative stress response (Kim et al., 2013) in *Arabidopsis*. The heterologous expression of exotic genes also effectively improves plant resistance to stress. When these TFs are overexpressed in plants, improved stress resistance has been detected. *STRESS RELATED NAC1* (*ZmSNAC1*), the ortholog of *RD26* in *Zea mays*, is strongly induced by low temperature, high salinity, drought stress and ABA treatment, and the expression improves osmotic stress tolerance in *Arabidopsis* and cotton (Lu et al., 2012; Shiriga et al., 2014).

A widely adopted strategy for identifying gene function is to introduce and express a gene in a model plant, which has a clearly different background that may influence the gene functions. However, to date, few reports have assessed the differences in phenotype and target gene expression between HE lines (with the heterologous expression of an ortholog) and OX lines (with an overexpressed native gene). How many genes change their expression levels in HE plants when a TF from a related species is introduced? This is a question that must be investigated. The choice of which homologous gene is important not only for genetic manipulation but also for understanding the interaction of the protein with DNA motif or other proteins.

In this study, an NAC TF from *E. salsugineum*, *EsNAC1*, the ortholog of *RD26* in *Arabidopsis*, was transformed into *Arabidopsis*. The heterologous lines of *EsNAC1* showed increased salt and oxidative stress resistance and accumulated much more starch and anthocyanidin, whereas vegetative growth was retarded, which was not identical to the RD26OX line. A genome-wide ChIP (chromatin immunoprecipitation analysis)-on-chip assay using an antibody against EsNAC1 revealed the target genes of EsNAC1, and the comparison of data from these genes to the data from an RD26OX line was performed. This investigation identified differences between the HE lines and OX lines and EsNAC1 showed the relatively differential effects in similar species.

## Materials and Methods

### Vector Construction and Transformation

**FIGURE 1:**
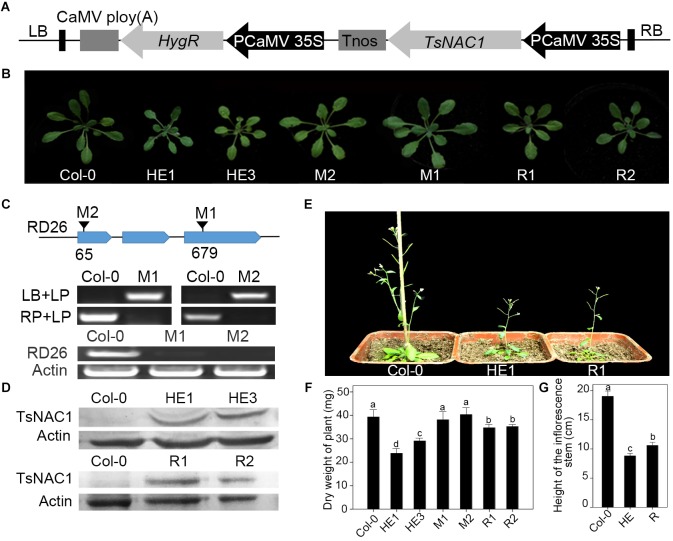
Heterologous expression of EsNAC1 retarded the growth of *Arabidopsis*. **(A)** The diagram for the T-DNA region of *EsNAC1* expression vector. **(B)** Rosettes of representative *EsNAC1* transgenic plants and mutants of RD26 4 weeks after planting. **(C)** The identification of homozygous T-DNA insert mutants. LP, LB and RP were primers provided on the T-DNA Primer Design page. The results of RT-PCR indicated the expression loss of RD26 in M1 and M2. **(D)** The western blot results of HE lines and Revertants (overexpressed *EsNAC1* in the T-DNA insertion mutants of RD26). **(E)** Phenotypes of *EsNAC1* transgenic plants after sowing 6 weeks in the medium of peat:vermiculite (2:1). **(F)** The dry weight of the whole plants. **(G)** The height of the inflorescence stems at 6 weeks. HE1, HE3: Different heterologous expression lines; M1, M2: Different T-DNA insert mutant lines; R1, R2: Different revertants. Values represent the mean ± SD, and those labeled with a letter are significantly different at *P* < 0.05 by Duncan’s test. Three biological replicates were in the experiment, with each biological replicate consisting of 20 plants.

The full-length cDNA of EsNAC1 from halophyte *Eutrema salsugineum* was ligated into the vector *pCAMBIA1300*, which contained hygromycin resistance as the selective marker. The vector was introduced into *Arabidopsis* (Col-0) by *Agrobacterium tumefaciens*-mediated transformation with strain GV3101 by the floral dip method to produce the HE1 and HE3 lines (Clough and Bent, 1998). Transformants were selected by planting seeds on MS plates containing 20 mg/l hygromycin B. The T2 homozygous transgenic plants were confirmed by genetic analysis of the segregation ratio of the generations. At the same time overexpressed *RD26* lines were produced with similar methods. T-DNA insertion mutants of RD26 (M1: SALK_072276 and M2: SALK_083756) were ordered from the *Arabidopsis* Biological Resource Center (ABRC). With reference to the primers provided on the T-DNA Primer Design page^1^, the homozygous mutants (M1 and M2) were identified by PCR and RT-PCR assays (**Figure 1C**). The homozygous mutants were transformed with the 35S::*EsNAC1* construct to produce the recovery lines R1 and R2. *Arabidopsis* plants were planted in a phytotron under the conditions of 22/16°C day/night temperatures, cool white light at an intensity of 5,000 lux, 16 h/8 h light/dark cycle, and with a relative humidity of 50%.

### Plant Growth and Stress Treatments

*Arabidopsis* seeds were planted in peat: vermiculite (2:1) and grown at 18°C under long day conditions (16 h light, 8 h dark) and with a relative humidity of 60%. For salt treatment, 4-week plants were watered with a 400 mM NaCl solution: 100 mL for each pot on the first day with a 20 mL addition every 3 days, maintaining a 200 mM NaCl concentration of the culture medium for 2 weeks. Plants were grown in the soil medium 4 weeks and exposed to drought stress, which was imposed by stopping the watering for 5 days. Uniform seedlings of transgenic and wild-type 2-leaf plants in each pot were sprayed with 0.1 mL of 0.1, 0.3, 0.5 or 1 mmol/l paraquat to mimic active oxygen stress treatment and then stained with NBT solution (Fryer et al., 2002). Germinated plants were grown on 1/2 MS medium with 0.15 μg/mL TM to mimic the ER stress.

### The Preparation of Polyclonal Antibody

The full-length coding sequence of *EsNAC1* was ligated into pGEX-4T-2, then the plasmid was transformed into *E. coli* strain BL21. The EsNAC1 expression strain was cultured, and protein expression was induced as described on pages 1221–1231 of “Molecular Cloning: a Laboratory Manual III.” Chelating Sepharose Fast Flow (GE Healthcare) were used for the purification of the soluble NAC-GST protein. The Abmart Company prepared the polyclonal antibody. The titer of the antibody was over 5 × 10^4^ IU. The effectiveness of the polyclonal antibody was tested with the Col-0 and the HE lines as materials (**Supplementary Figure S5**).

### Binding Activity Assay of EsNAC1 in the Yeast System

The coding sequence of *EsNAC1* was obtained by PCR, and the product was digested with EcoRI/PstI and then connected to the pGADT7-AD vector, which contained the GAL4 active domain. The promoters of candidate target genes were cloned into pLacZi. Two types of the plasmids were transformed into the yeast strain YM4271 (Invitrogen) in pairs. The β-galactosidase activity was examined by X-gal staining and measured as described in the Yeast Protocols Handbook (Clontech) using ONPG (*o*-nitrophenyl-β-D-galactopyranoside) as the substrate.

### ChIP-on-Chip and ChIP-qPCR

The details of ChIP were performed as described by Kaufmann et al. (2010) using 3-week-old plants of HE1 line with two biological replicates. The labeling of probes (cell extract DNA (input) and ChIP DNA were labeled with Cy3- and Cy5-labeled random 9-mers, respectively, and a dye-swap was performed in the experiment) and the chip hybridization were accomplished by Shanghai Kang Cheng Biological Technology Ltd. The software used to analyze the chip data was provided by Roche. The chip used was the 2006-11-01_ATH6_min_promoter microarray (Roche), which contains approximately 38,500 probes and covers all the promoter regions that have been identified in *Arabidopsis* (i.e., approximately −1,200 bp to +300 bp surrounding the TSSs). Raw data were extracted as pair files by NimbleScan software. Log_2_-ratio data were calculated and scaled by subtracting the bi-weight mean. At the same time, false discovery rate (FDR) for each peak whose signals were above the specified cutoff values were computed using a permutation-based algorithm. The cutoff values are a percentage of a hypothetical maximum, which is the mean + 6 [standard deviation]. The ratio data is then randomized 20 times to evaluate the probability of “false positives.” Each peak is then assigned a FDR score based on the randomization. And the number of false peaks will increase with the improvement of FDR. The lower the FDR score, the more likely the peak corresponds to a protein binding site. And the peaks with FDR score ≤ 0.05 are considered high-confidence binding sites in this study. The raw data were uploaded to the data sharing platform figshare^2^. **Supplementary Table S2** showed all the candidate target genes of EsNAC1 in *Arabidopsis*.

The 3-week plants of HE1 line were sampled for the ChIP-real-time qPCR assay with three biological replicates. All samples were diluted to 10 ng/μl, and the reaction mixture was as follows: SYBR^®^ Premix Ex Taq^TM^ (2x), 5 μl; PCR forward primer (10 μM), 0.2 μl; PCR reverse primer (10 μM), 0.2 μl; DNA template, 1 μl; and distilled deionized H_2_O up to 10 μl. The primers used to amplify the enriched regions of the target sequences are listed in **Supplementary Table S3**. And all the data were normalized by the Input (without the immunoprecipitation with the antibody of EsNAC1) level.

### Determination of Photosynthetic Pigment, Proline, and Starch Contents

Pigments was extracted with 95% alcohol under dark conditions, and the contents of Ch a, Ch b, and Car were quantified spectrophotometrically, according to the method of Wellburn (Wellburn and Lichtenthaler, 1984).

Free proline contents were measured using L-proline as the standard according to Bates et al. (1973). Leaf samples (0.2 g) were homogenized in 10 mL of 3% (w/v) aqueous sulphosalicylic acid and filtered. Two milliliters of the solution was then mixed with 2 mL of acid ninhydrin and 2 mL of glacial acetic acid in a test tube and incubated in a 100°C water bath for 30 min. The free proline content of the solution was finely extracted with 4 mL of toluene. The absorbance was recorded at 520 nm, and the proline concentration was determined as μmol/g fresh weight using a standard curve.

Starch was determined with reference to Rojas-González et al. (2015).

### Chlorophyll Fluorescence Determination

The determination of *F*_0_ and *F*_v_/*F*_m_ was executed with a pulse modulation chlorophyll fluorescence spectrometer (LI-COR Inc., Lincoln, NE, United States). Plants were dark-adapted for 20 min before measurement.

### Quantitative RT-PCR Analysis

The 3-week-old wild type, HE1 line and M2 line plants were executed for RT-qPCR with each three biological replicates of them. Total RNA was extracted with TRIzol (TAKARA, Japan) reagent. Total RNA, 500 ng, was used for reverse transcription with a Transcriptor First Strand cDNA Synthesis Kit (TAKARA, Japan). The cDNA was diluted 10-fold and then used as the template for real-time RT-PCR. The RT-PCR reaction mixture was as follows: SYBR Premix Ex Taq (2x), 5 μl; PCR forward primer (10 μM), 0.2 μl; PCR reverse primer (10 μM), 0.2 μl; cDNA template, 1 μl; and distilled deionized water up to 10 μl. The thermocycling conditions were as follows: 3 min at 95°C, followed by a total of 40 cycles of 15 s at 95°C, 30 s at 58°C, and 30 s at 72°C.

## Results

### EsNAC1 Heterologous Expression Inhibited the Growth of *Arabidopsis*

To identify the function of EsNAC1, HE1&3 [35S promoter-driven EsNAC1 (**Figure 1A**) in Col-0], M1&2 (Homozygous mutants of RD26, **Figure 1C**) and R1&2 [35S promoter-driven EsNAC1 in M1&2 (**Figure 1D**)] were identified. Compared with wild-type Col-0 plants, mutants M1 and M2 did not show significant differences in organ size (**Figure 1B**), whereas the shoot size in the HE lines was notably smaller than that of Col-0 (**Figure 1B**), with 63.1% dry weight of Col-0 plants (**Figure 1F**). For the revertants R1 and R2, the shoot size was smaller than that of Col-0 but larger than that of the HE lines (**Figure 1B**), with 74.5% the dry weight of Col-0 on average (**Figure 1F**). During the mature stage, the inflorescence stems of revertants were much shorter than those of Col-0 but 1.2-fold longer than those of the HE lines (**Figures 1E,G**). In summary, the heterologous expression of EsNAC1 in *Arabidopsis* stunted the plants; this phenotype is highly consistent with that of transgenic 35S::RD26 plants in *Arabidopsis* (Ye et al., 2017), and these inhibitory effects were relatively weak in the R lines. These results implied that EsNAC1 had a similar function to that of RD26 on growth in *Arabidopsis*.

### Heterologous Expression of EsNAC1 Improved Plant Abiotic Stress Resistance

**FIGURE 2:**
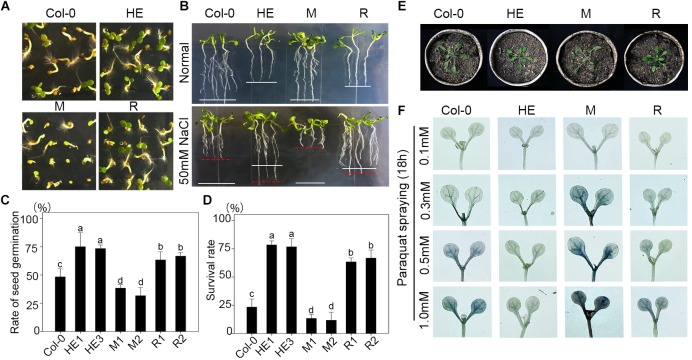
Overexpressed *EsNAC1* improved the salt and oxidative stress resistance of *Arabidopsis*. **(A)** Phenotypes of seedlings from Col-0 and HE, M and R lines after germinating on 1/2 MS-agar with 100 mM NaCl for 3 days. **(B)** Phenotypes of plants from Col-0 and HE, M and R lines after germinating on 1/2 MS-agar for 10 days or 50 mM NaCl for 15 days. White lines denote the average length of plants under the normal condition; red lines denote the average length of plants under salt stress. **(C)** The statistical results for rate of seed germination on 1/2 MS-agar with 100 mM NaCl after sowing for 5 days. **(D)** The statistical results of the survival rate of 4-week-old plants after 200 mM NaCl treatment for 7 days. **(E)** Phenotypes of 4-week-old plants from Col-0 and HE, M and R lines after watering was stopped for 5 days. **(F)** The NBT staining results after treatment with different concentrations of paraquat for 18 h. Values represent the mean ± SD, and those labeled with a letter are significantly different at *P* < 0.05 by Duncan’s test. Three biological replicates were in the experiment, with each biological replicate with four dishes or 16 plants.

When sowing seeds of transgenic and wild-type plants on 1/2 MS medium with 100 mM NaCl, the germination rate of the HE lines was 1.54-fold higher than that of the wild type and 2.27-fold higher than that of the M lines (**Figures 2A,C**), and the growth of the HE and R lines on 1/2 MS medium with 50 mM NaCl was much better than that of Col-0 and the mutants (**Figure 2B**). When plants grew in a medium consisting of peat:vermiculite (2:1, v/v) for 4 weeks under normal conditions, they then were subjected to salt stress, and the heterologous expression of EsNAC1 improved plant tolerance of high salt to 200 mM NaCl, with about 80% of the plants survived after salt stress treatment, which was substantially higher than the 25% and 15% survival rates observed in Col-0 and the M lines (**Figure 2D**). These results indicated that the heterologous expression of EsNAC1 complemented the function of RD26 in the mutants and remarkably improved the salt stress tolerance of Col-0. The proline contents in the HE and R lines were higher than that of Col-0 and the mutants under normal and salt stress conditions. On a medium with 200 mM NaCl, the HE lines accumulated much more proline than that of the R lines did (**Supplementary Figure S1**). Furthermore, 4-week-old plants of the HE and R lines showed a better state than that of the Col-0 and the M plants after stopping watering for 5 days (**Figure 2E**). When the seedlings were sprayed with different concentrations of paraquat, the HE and R lines showed higher oxidative stress resistance than that of the Col-0 and the M lines. Compared with the R lines, the HE lines showed higher resistance when the concentration of paraquat increased to 1.0 mM (**Figure 2F**).

A wide range of detrimental cell environments can induce endoplasmic reticulum (ER) stress. To understand the effects of the heterologous expression of *EsNAC1* on ER stress, the seeds of Col-0 and the HE, M and R lines were germinated on 1/2 MS medium supplemented with 0.15 μg/mL tunicamycin (TM). The M line showed a significantly reduced rate of germination, and many seedlings had poorly developed cotyledons, whereas the Col-0 or the R lines had better seedlings, the proportion of seedlings with poorly developed cotyledons was higher than that of the HE line (**Supplementary Figure S2**). These results implied that the heterologous expression of *EsNAC1* affected cell response to ER stress and alleviated the detrimental effects of the unfolded protein response (UPR).

### Identification of the Target Genes of EsNAC1 in *Arabidopsis*

**FIGURE 3:**
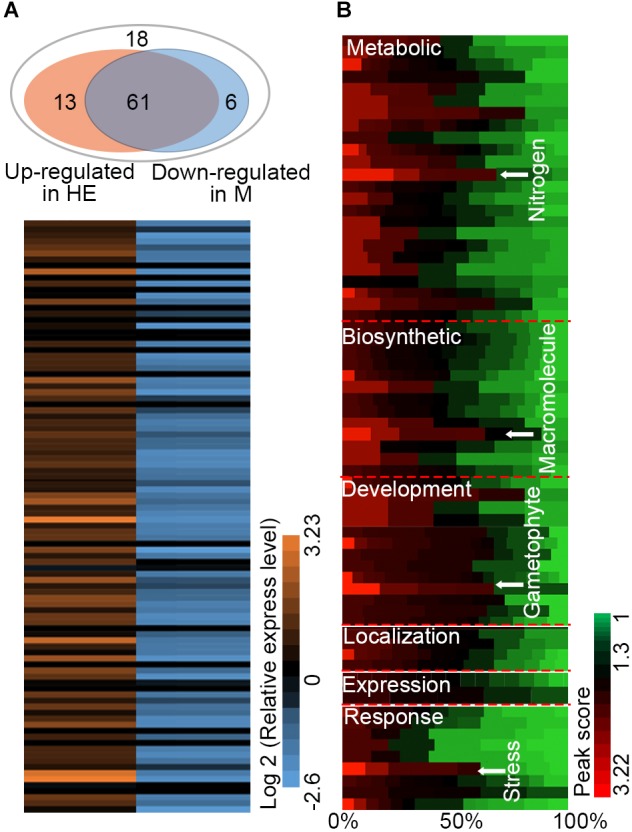
Candidate target genes of EsNAC1 in *Arabidopsis*. **(A)** The expression analysis of 98 EsNAC1 candidate target genes (FDR ≤ 0.05, Peak score [–log_10_ (*P*-value) ≥ 1.3] in the HE line compared with that of corresponding genes in the M line. HE1 line and M2 line were sampled with three biological replicates for RT-qPCR. Sixty-one genes that increased expression [relative expression level (REL, log_2_ ratio) ≥ 1] in the HE line showed reduced expression (REL ≤ –1) in the M line. All data were normalized to the target gene expression level in Col-0. **(B)** Binding situation of EsNAC1 quantified by the percent of genes (*x*-axis) in different gene ontologies (*y*-axis) with the color of each square mapped to the peak scores. Peak score: –log_10_ (*P*-value) for each basepair through local Poisson test, which means the ChIP signal at each basepair will be tested against the whole genome background of *Arabidopsis*, and the ratio of Peak score _IP_/Peak score _input_ was showed.

**FIGURE 4:**
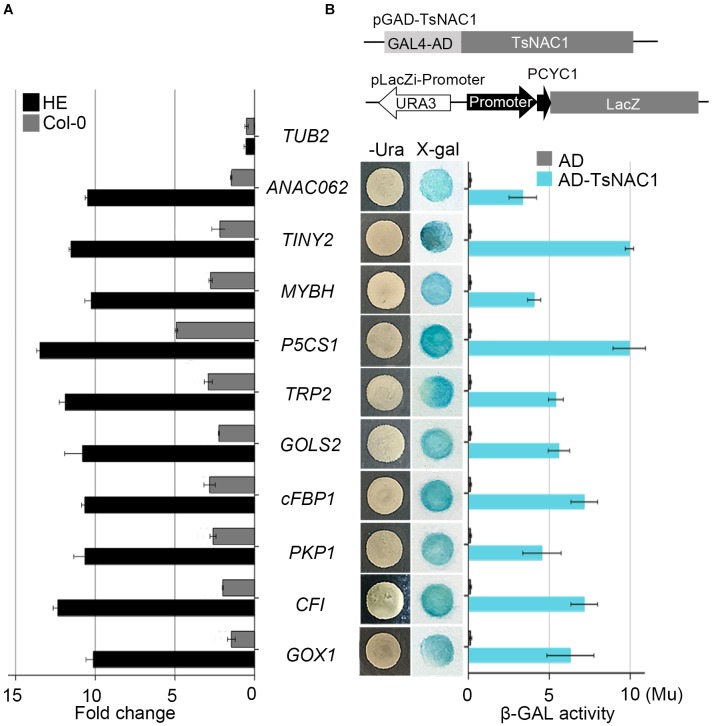
Validation of candidate target genes. **(A)** Binding of EsNAC1 to the promoter of candidate target genes as determined by ChIP-qPCR. The rosette leaves of 3-week-old plants from the two HE lines were used as materials and immunoprecipitated with EsNAC1 antibody and protein A agarose beads. Negative control reactions were performed in parallel with Col-0. **(B)** Candidate target promoter fragments were connected into pLacZi and transformed into the pAD-GAL4-*EsNAC1* YM4271 strain. X-gal staining determined β-galactosidase activities. Measures of β-gal activity were with ONPG as the substrate. β-gal units (Mu), 1,000 × OD_574_/(t × V × OD_600_); t, elapsed incubation time (min); V, volume of culture (mL). All bars represent the mean ± SD, with three biological replicates in the experiment.

The HE lines of *Arabidopsis* were used as the materials for ChIP-on-chip assay and 98 target genes of EsNAC1 were identified with an FDR ≤ 0.05 and a peak score [−log_10_ (*P*-value)] ≥ 1.3. To test the veracity of the results, a subset of the candidate downstream genes with high peak scores were selected, and their expression levels were examined in the wild type, HE1 line and M2 line by RT-PCR (**Figure 3A**). Based on the results, of the 98 selected members, more than 75.5% (74) of the genes increased their expression more than twofold in the HE line compared with that of the wild type, whereas in the M line, >82% (61 of 74) of the upregulated genes in the HE line showed lower expression levels compared with the wild type (**Figure 3A**). These results demonstrated that most of the candidate target genes from the ChIP-on-chip assay were regulated by the heterologous expression of *EsNAC1* or may by its ortholog RD26. Primary Gene Ontology (GO) annotation analysis of the candidate target genes of EsNAC1 in *Arabidopsis* with peak score ≥ 1 (174 genes) indicated that EsNAC1 played roles in biological processes, such as the regulation of metabolism, biosynthesis, plant development, gene expression, and response to stress. In the term of molecular functions, enrichment was in TF activity and acid-amino acid ligase activity. A follow-up functional enrichment analysis of GO (*y*-axis) against the percent of genes (*x*-axis) with the color squares representing the peak score indicated that EsNAC1 might play crucial roles in the nitrogen compound metabolic process (GO:0006807), macromolecule biosynthetic process (GO:0009059), gametophyte development (GO:0048229), and response to stress (GO:0006950) (**Figure 3B** and **Supplementary Figure S3**). Based on the peak score (>1.71) and log_2_ values (relative expression level in HE lines to Col-0) in the HE lines, 10 of the genes in the enriched GO subsets were selected for further analysis. ChIP-qPCR results indicated that EsNAC1 could specifically recognize and bind their promoters in *Arabidopsis* (**Figure 4A**). Then, their promoter regions were cloned into the pLacZi vector for yeast one-hybrid assays. The X-gal staining results showed that EsNAC1 targeted these promoter regions (**Figure 4B**).

### EsNAC1 Targeted Genes That Play Crucial Roles in Plant Resistance to Abiotic Stress

Analysis of the EsNAC1 ChIP-on-chip data identified several TFs related to environmental stress tolerance, including *NAC DOMAIN CONTAINING PROTEIN 62* (*ANAC062*, AT3G49530), *Integrase-type DNA-binding protein* (*TINY2*, *AT5G11590*) and *MYB HYPOCOTYL ELONGATION-RELATED* (*MYBH*, *AT5G47390*), as the candidate target genes of EsNAC1. EsNAC1 upregulated the expression levels of *ANAC062*, *TINY2* and *MYBH* (**Table 1**). Moreover, some genes involved in abiotic stress resistance were also upregulated by EsNAC1 in the HE lines (**Table 1**), including *DELTA1-PYRROLINE-5-CARBOXYLATE SYNTHASE 1* (*P5CS1*, *AT2G39800*), involved in the accumulation of proline (Leprince et al., 2014; Bhaskara et al., 2015); *GALACTINOL SYNTHASE 2* (*GOLS2*, *AT1G56600*), which is a key enzyme in the biosynthesis of raffinose family oligosaccharides (RFOs) that participate in environmental stress adaptation (Kim et al., 2012; Song et al., 2016); and *TRYPTOPHAN BIOSYNTHESIS 2* (*TRP2*, *AT5G54810*), which plays a role in tryptophan biosynthesis and salt stress tolerance (Zhao et al., 1998). The binding of EsNAC1 to the promoters of these genes was determined by the ChIP-qPCR assay (**Figure 4A**) and one-hybrid yeast assay (**Figure 4B**).

**Table 1:** Candidate targeted genes of EsNAC1 in *Arabidopsis*.

AGI Number^a^	REL(+)^b^	REL(−)^c^	Peak score^d^	Functional description
AT4G34640	2.21	0.36	3.22	Squalene synthase 1 (SQS1)
AT3G54050	3.75	0.31	2.92	Fructose-1,6-bisphosphatase (cFBP1)
AT5G47390	7.85	0.37	2.92	Myb-like transcription factor protein (MYBH)
AT3G49530	9.26	0.25	2.7	NAC domain containing protein 62 (ANAC062)
AT5G11590	6.43	0.23	2.55	Integrase-type DNA-binding protein (TINY2)
AT3G22960	9.43	0.36	2.09	Pyruvate kinase family protein (PKP1)
AT3G14420	8.54	0.34	2.09	Aldolase-type TIM barrel family protein (GOX1)
AT3G02630	5.32	0.21	2	Plant stearoyl-acyl-carrier-protein desaturase family protein
AT5G54810	9.39	0.24	2	Tryptophan synthase beta-subunit 1 (TRP2)
AT3G24800	2.56	0.37	1.93	Proteolysis 1
AT3G49370	3.23	0.29	1.93	Calcium-dependent protein kinase (CDPK)
AT2G39800	6.75	0.45	1.74	Delta1-pyrroline-5-carboxylate synthase (P5CS1) 90 1 (P5CS1)
AT3G55120	4.27	0.32	1.73	Chalcone-flavanone isomerase family (CFI)
AT3G32029	3.12	0.35	1.73	Transposable element gene
AT5G63690	4.71	0.33	1.72	Nucleic acid-binding, OB-fold-like protein
AT4G34200	5.25	0.32	1.72	D-3-phosphoglycerate dehydrogenase (EDA9)
AT1G56600	3.54	0.27	1.72	Galactinol synthase 2 (GOLS2)
AT3G09070	2.26	0.42	1.71	Protein of unknown function (DUF740)

*AGI^a^, Arabidopsis Genome Initiative. REL (+)^b^, the relative expression level in HE lines. REL (−)^c^, the relative expression level in M lines. All the data was normalized by the target gene’s expression level in Col-0. Peak Score^d^, the Peak Score referenced to ChIP-on-chip.*

EsNAC1 also targeted positive regulators of oxidative stress resistance. A crucial gene for the accumulation of purple anthocyanins in response to reactive oxygen stress is *CHALCONE FLAVANONE ISOMERASE* (*CFI, AT3G55120*). One of the glycolate oxidases was *GLYCOLATE OXIDASE 1* (*GOX1, AT3G14420*), which modulates reactive oxygen signal transduction. The ChIP-qPCR analyses (**Figure 4A**) indicated that these genes were both targeted by EsNAC1. The quantitative RT-PCR results indicated that the expression levels of both *CFI* and *GOX1* were upregulated (**Table 1**) in the HE lines. These data suggested that the role of *CFI* and *GOX1* in improving plant reactive oxygen stress was directly regulated by EsNAC1.

Overall, these results demonstrated that EsNAC1 regulated the expression of resistance genes in the response to environmental stresses and increased the resistance of plants to abiotic stresses.

### EsNAC1 Was Involved in the Regulation of Carbohydrate Accumulation During the Light/Dark Cycle

**FIGURE 5:**
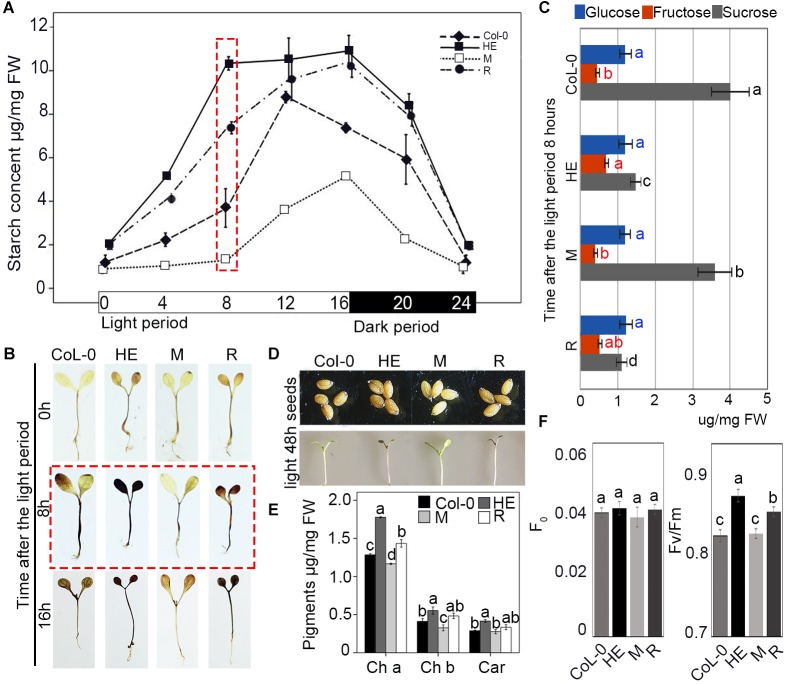
EsNAC1 was involved in the accumulation of starch and pigment. **(A)** Changes in starch content in Col-0 and HE, M and R lines. The rosette leaves were collected at 0, 4, 8, 12, 16, 20 (4 h after dark period), and 24 h (8 h after dark period) in the light/dark cycle. **(B)** Staining results with Lugol’s solution at 0, 8, and 16 h of the light period. **(C)** The concentrations of glucose, fructose, and sucrose in Col-0 and HE, M and R lines at 8 h of the light period. **(D)** The phenotypes of seeds and seedlings 5 days after sowing after 48 h of light. HE and R lines showed the accumulation of anthocyanin. **(E)** Contents of chlorophyll *a* and *b* and carotenoids in Col-0 and HE, M and R lines. **(F)** *F*_0_ and *F*_v_/*F*_m_ in Col-0 and HE, M and R lines. Fv, maximal fluorescence; Fm, variable fluorescence. Values represent the mean ± SD, and those labeled with a letter are significantly different at *P* < 0.05 by Duncan’s test. Three biological replicates were in the experiment, with each biological replicate with 20 or 8 (for *F*_0_ and *F*_v_/*F*_m_, respectively) plants.

To explore the mechanism of growth retardation in the HE and R plants, the starch content in Col-0 and the HE, M and R lines was analyzed every 4 h in a circadian rhythm of 16 h light and 8 h dark. During the light period, all lines accumulated starch. After 8 h of light, the HE and R lines displayed 2.8-fold and 1.9-fold higher starch contents than that of Col-0, respectively, whereas the starch content in the M line was less than half that of Col-0 (**Figure 5A**). When illuminated for 12 h, the starch contents of Col-0, the HE and R lines increased to the higher levels with less disparity, because the starch accumulation operated with different accumulation rates. In the HE and R lines starch accumulation continued through 16 h of illumination, whereas in the Col-0 plants, starch content rapidly fell in the 12–16 h. M line showed faster starch accumulation in the 8–16 h of the illumination (**Figure 5A**). Staining with Lugol’s solution confirmed the higher starch accumulation in the HE and R lines than that in Col-0 (**Figure 5B**). EsNAC1 undoubtedly enhanced starch accumulation during the light period. In dark period, all lines decreased their starch contents until near zero levels at the end of the period, which showed degradation rates were related to starch contents (**Figure 5A**). These results implied that the HE and R lines accumulated excess starch in the light that was quickly exhausted during the dark period, which indicated that EsNAC1 modified carbohydrate metabolism during the light/dark cycle. By the determination of the levels of glucose, fructose, and sucrose in the plants of the different lines, we found fructose and glucose were maintained at similar levels in the different lines and the concentration of sucrose was much higher than that of glucose or fructose in the plants of Col-0 and M lines after 8 h of illumination, and the sucrose in the HE and R lines was significant lower when compared to Col-0 (**Figure 5C**). The decrease sucrose contents and the steady levels of glucose were consistent with the accumulation of starch, which supported that EsNAC1 was involved in the regulation of sugar anabolism.

Chloroplastic fructose-1,6-bisphosphatase (*cFBP1, AT3G54050*) is a key enzyme in the Calvin–Benson pathway; the lack of cFBP1 in the *Arabidopsis* mutant *cfbp1* causes a decrease in rosette size and a reduced rate of photosynthesis, a decrease in the content of soluble sugars, and reduced starch accumulation (Rojas-González et al., 2015). *PLASTIDIAL PYRUVATE KINASE 1* (*PKP1*, *AT3G22960*) encodes a chloroplast pyruvate kinase alpha subunit. According to the ChIP-qPCR and the one-hybrid yeast assay, EsNAC1 bound to the promoters of these two genes (**Figures 4A,B**). EsNAC1 upregulated the expression levels of cFBP1 and PKP1 (**Table 1**), leading to the inference that EsNAC1 increased starch accumulation by positively regulating the expression of *cFBP1* and *PKP1*.

### Differences in the Regulatory Networks of EsNAC1 and RD26

**FIGURE 6:**
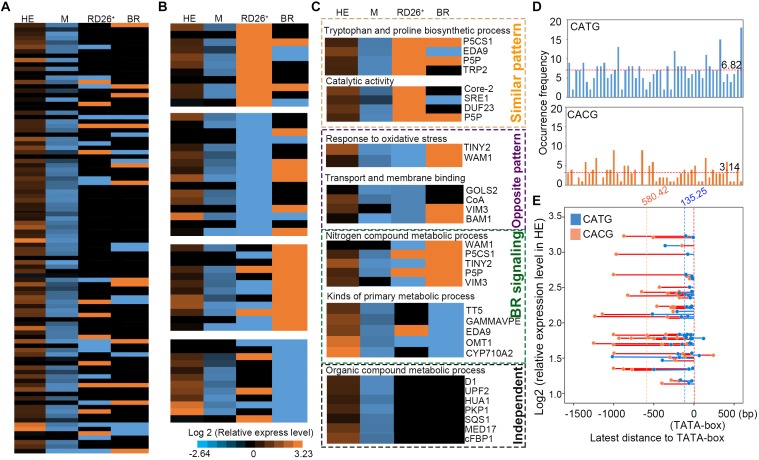
Comparison of expression levels between the target genes of EsNAC1 in HE lines and up- or down-expressed genes in 35S:RD26 lines and the BR treatment of *Arabidopsis*. **(A)** The expression levels of genes were ranked referenced to the peak score in Chip-on-chip. HE, heterologous expression lines of *EsNAC1*; M, the mutant lines of RD26; RD26^+^, the 35S::RD26 lines of Col-0 from Ye et al. (2017); BR, plants with the brassinosteroid treatment from Ye et al. (2017) **(B)** Genes were repermutated with reference to the positive- or negative-correlated expression pattern. **(C)** The orange dot box indicates genes with a similar expression pattern to that of the RD26OX line; the purple dot box marks genes with the opposite expression pattern compared with that of the RD26OX line; the green dot box labels genes for which expression changed after the BR treatment; the black dot box shows genes regulated by EsNAC1 independent of the BR signaling pathway and different from those of the RD26OX line. All data were normalized to the gene expression level in Col-0. **(D)** Occurrence frequency of CATG and CACG in promoter regions shown in a. Red dotted lines represent the mean occurrence frequency. **(E)** Distances between the latest CATG (blue) or CACG (orange) to the TATA-box against with the relative expression level of the gene in HE lines. Blue or orange dotted lines represented the means of these latest distances.

**FIGURE 7:**
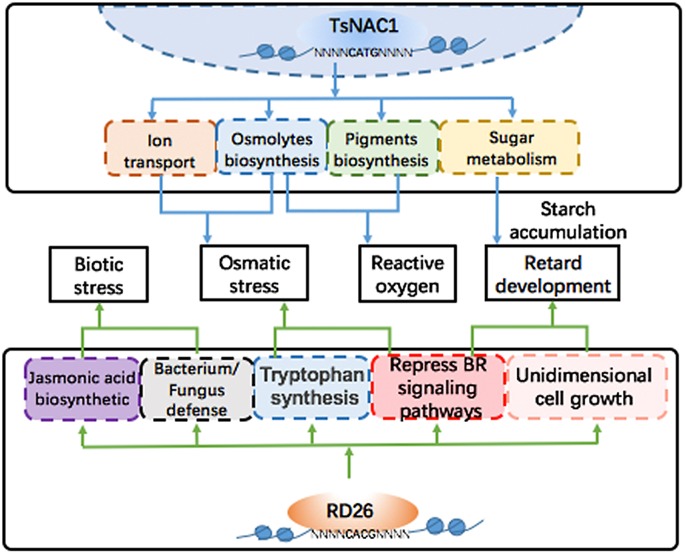
Regulated networks of EsNAC1 and RD26. EsNAC1 improved the osmotic stress and the reactive oxygen stress tolerance of plants via regulating the transport of ions and the biosynthesis of pigments and osmolytes and retarded the development of plants via regulating the sugar metabolism and the accumulation of starch. By contrast, the overexpression of RD26 retarded plant development via repressing genes of the BR signaling pathways and unidimensional cell growth. Additionally, RD26 could regulate the biosynthesis of jasmonic acid to improve biotic stress tolerance but showed no genes for reactive oxygen stress tolerance.

We compared the RNA-seq results of the RD26OX (35S::RD26) line from Ye et al. (2017) and the results from the HE lines of EsNAC1 and the M lines (**Figure 6**) and found differences between the regulatory networks of EsNAC1 and RD26 (**Figure 7**).

The heterologous expression of EsNAC1 increased high salinity stress tolerance up to 200 mM NaCl in *Arabidopsis*, which was higher than that of the RD26OX line. The expression of RD26 is induced by drought and high salinity (Fujita et al., 2004), and several stress-inducible genes involved in the synthesis of tryptophan and proline were upregulated in the RD26OX line, similar to the results in the HE lines. Additionally, some genes encoding enzymes with catalytic activity, such as C*ore-2/I-branching beta-1,6-N-acetylglucosaminyltransferase* (*Core-2*, *AT1G62305*), *NAD(P)-binding Rossmann-fold superfamily* (*SRE*, *AT1G52340*) and *Pyridoxal-5′-phosphate-dependent enzyme family protein* (*P5P*, *AT5G28237*) (**Figure 6**), showed similar expression patterns in the HE lines and the RD26OX line. However, some genes, such as *TINY2* and *WALDMEISTER 1* (*WAM1*), that responded to oxidative stress, and genes that encoded proteins with transport or membrane binding abilities [*GOLS2*, *SUCCINYL-COA LIGASE* (*CoA*, *AT5G23250*), *VIM3*, *BETA-AMYLASE 1* (*BAM1*, *AT5G65700*)] showed opposite expression patterns in the HE lines and the RD26OX line (**Figure 6**). The upregulated expression of these genes in the HE lines and their effects on oxidative stress response and tolerance might contribute to the increase in adaptability and tolerance to high salinity of the HE lines (**Figure 2**).

As shown by the expression pattern of RD26 in the *Arabidopsis* eFP Browser, RD26 is induced by certain biotic stress treatments. In the RD26OX line, genes that participated in the biosynthesis of jasmonic acid and in bacterial and fungal defense were clearly either upregulated or repressed. However, only a fraction of these genes was found among the target genes of EsNAC1 (data not shown). Furthermore, genes involved in the organic compound metabolic process, such as *3 beta-hydroxysteroid -dehydrogenase/decarboxylase isoform 1* (*D1*, *AT1G47290*), *Floral homeotic protein* (*HUA1*, *AT3G12680*), *PKP1*, *Squalene synthase 1* (*SQS1*, *AT4G34640*), *RNA polymerase II transcription mediators* (*MED17, AT5G20170*) and *cFBP1*, and genes involved in brassinosteroid (BR) signaling showed different expression patterns in RD26OX compared with those in the HE lines (**Figure 6C**). The repression of amylases generated abnormal starch accumulation in the light period, which might be an important factor for the retardation of growth. We found that the expression level of types of amylases, such as α-amylase-like 3 and β-amylases 4, 5, and 9, was obviously downregulated in the HE lines (**Supplementary Table S1**), although none of them were identified as the direct downstream target gene of EsNAC1 in *Arabidopsis*, while in RD26 OX plants only β-amylase 9 was downregulated (Ye et al., 2017). These results indicated EsNAC1 might control the expression of amylases indirectly and could explain the accumulation of starch and anthocyanins in HE lines (**Figures 5A,B**). Recent studies show that the downregulation of genes in the BR signaling pathways and unidimensional cell growth play crucial roles in the retardation of growth in the *Arabidopsis* overexpression lines of RD26 (Ye et al., 2017). Only some of these genes were identified in the ChIP-on-chip results for HE lines. By comparing the RNA-seq results for RD26OX (Ye et al., 2017) and our real-time RT-PCR results, the regulatory networks of EsNAC1 and RD26 were different in *Arabidopsis* (**Figures 6**, **7**), and their target genes were not identical.

To verify EsNAC1 and RD26 had different target genes, the promoter regions (approximately −2,000 bp to 0 bp to ATG) of 50 candidate downstream genes [61 candidate downstream target genes (**Figure 3B**) of EsNAC1 were referenced on the ChIP-on-chip, excepted 11 without specific promoter regions such as transposons] were exported with the web service of Gramene (**Supplementary Figure S4**). The distributions of CATG (the binding motif of EsNAC1) and CACG (the binding motif of RD26) in these promoter regions were different, and the average occurrence frequency of CATG was 6.82 and that of CACG was 3.14 (**Figure 6D**). When the latest distance between CATG or CACG to the TATA-box to plot were matched to the relative expression level of the gene in HE (**Figure 6E**), it was clear that CATG was much closer than CACG to TATA-box in promoter regions of candidate downstream genes of EsNAC1. Usually, the closest TF binding site to the TATA-box was the principal motif for the control of the gene expression, we inferred EsNAC1 plays crucial role in the regulation of transcription for the majority of these genes. It could be explicit that the distinctions of the target genes caused the differences between the HE and RD26OX lines in *Arabidopsis*, which was related to the distribution discrepancy of the binding motifs of EsNAC1 and RD26.

## Discussion

### EsNAC1 Regulated Abiotic Stress Resistance in *Arabidopsis*

Heterologous expression of the NAC TF EsNAC1 in *Arabidopsis* improved abiotic stress resistance and retarded plant growth. By utilizing ChIP-on-chip to identify the binding regions of a TF on the chromosomes and to classify the possible downstream genes is a commonly accepted method for the discovery of target genes. In this study, 98 candidate target genes of EsNAC1 were identified, and among them, some were involved in developmental process, metabolic process, biosynthetic process, response to stress and gene expression, while others played roles in transcription regulation, oxidoreduction activity and acid-amino acid ligase activity (**Figure 3C**). Of the candidate target genes (**Table 1**), ANAC062 has roles in abiotic stress responses, such as high salinity in seed germination (Piljoon and Chungmo, 2010) and drought stress (Mi et al., 2012; Yang et al., 2014), and mediates ER stress in *Arabidopsis*, in which it regulates the expression of several UPR genes such as *LUMINAL BINDING PROTEIN2* (*BiP2*) (Yang et al., 2014). Overexpression of BiP2 alleviates the accumulation of Cd^2+^ to relieve ER stress-induced cell death (Xu et al., 2013) and has a role in osmotic stress tolerance. TINY2 is a member of the DREB subfamily A-4 in the AP2/ERF TF family, and the expression of TINY2 is induced by drought and salt stress treatment (Wei et al., 2005). Some TINY subfamily members, such as *ATERF019*, *FYF UP-REGULATING 321 FACTOR 1* (*FUF1*) (Ma et al., 2006), *HARDY* (Abogadallah et al., 2011) and *ETHYLENE AND SALT INDUCIBLE 2* (*ESE2*) (Zhang et al., 2011), respond to salt stress treatment, and *HARDY* improves the salt and drought tolerance of *Arabidopsis* by reducing the uptake of Na^+^ (Abogadallah et al., 2011). *MYBH* responds to osmotic stresses such as salt and drought stress in *Arabidopsis* (Rasheed et al., 2016). *MYB* family proteins, such as *MYB2*, *21*, *108*, *112*, and *116*, are among the target genes of *RD26* (Hickman et al., 2013) and are involved in salt and dehydration tolerance (Yang et al., 2012; Wang et al., 2015). Previous studies of rice show that *OsMYBc* regulates the expression of *K*+ TRANSPORTER (OsHKT1) to reduce the accumulation of Na+ and improve salt stress resistance in rice (Zhang et al., 2015). In the HE lines, the expression of EsNAC1 upregulated the expression of ANAC062, TINY2, GOLS2 and P5CS1 genes, which increased abiotic stress resistance. In addition to the above genes, some well-studied TFs such as VIM1 and 3 (AT1G57820 and AT5G39550) and MYB95 (AT1G74430) were also upregulated in the HE lines (**Supplementary Table S1**). VIM proteins regulate transcription via modulating the methylation of DNA (Kim et al., 2014), and overexpressing VIM1 leads to an inhibition of root growth (Liu et al., 2010). Other genes, such as *MRS2-7* (magnesium transporter 7), which affects the transportation of Mg^2+^ similar to MRS2-2 (Gebert et al., 2009), and the H^+^ transporter *VACUOLAR H*^+^*-PUMPING ATPASE C1* (*VHA-C1*) (Dow et al., 1992), with cation transmembrane transporter activity, were also upregulated in HE lines (**Supplementary Table S1**). Construction of a new ion homeostasis is very important for plant resistance to osmotic stress, by altering ion transport, such as by overexpressing the H^+^-pyrophosphatase AVP1, which improves the salt stress tolerance of plants (Li and Gaxiola, 2005). Therefore, the heterologous expression of EsNAC1 improved abiotic stress resistance in *Arabidopsis* by regulating the expression of downstream target genes.

The accumulation of osmolytes, such as proline, raffinose and tryptophan, has an important role in the osmotic stress adjustment of plants. This study identified that *P5CS1* and *GOLS2* were directly upregulated by EsNAC1. *P5CS1* is the rate-limiting enzyme in the biosynthesis of proline (Yoshiba et al., 1999). The mutant *p5cs1-1* is sensitive to high salinity and accumulates less proline in response to salt stress than that of Col-0; this mutation is also correlated with a reduced transcript level of *P5CS1* (Székely et al., 2008; Leprince et al., 2014; Ibragimova et al., 2015). The content of proline in the HE and R lines was higher than that in Col-0 and the mutants under both normal and salt stress conditions (**Supplementary Figure S1**). *GOLS2* encodes a galactinol synthase and its overexpression improves salt, oxidative and drought stress tolerance in *Arabidopsis* and rice (Himuro et al., 2014; Selvaraj et al., 2017). The overexpression of *GOLS2* also increases grain yield by regulating the accumulation level of galactinol (Guo et al., 2010; Selvaraj et al., 2017).

A previous study has indicated that the accumulation of anthocyanins contributes to oxidative stress resistance (Meng et al., 2014). Anthocyanins are phenolic compounds belonging to a group of molecules called flavonoids, and their accumulation requires flavanones. CFI catalyzes the conversion of chalcones into flavanone, and *tt5-1* (the mutant of CFI) has yellow seeds and is sensitive to chlorophyll breakdown (Qin et al., 2011). The HE and R lines had brown seed coats and obvious anthocyanin accumulation (**Figure 5D**), in addition to relatively high contents of chlorophyll a (Ch a), Ch b, and a high Ch a/Ch b ratio (**Figure 5E**); a higher quantum yield (*F*_v_/*F*_m_) occurred in the stem and leaves of plants after exposure to light for 48 h (**Figures 5D,F**). *GOX1* (*GLYCOLATE OXIDASE 1*), a candidate target gene of EsNAC1, has positive effects on oxidative stress resistance (Saji et al., 2017), and the T-DNA insertion mutant lines show decreased chlorophyll (Saji et al., 2017) and sensitivity to reactive oxygen species, and have similar phenotypes to the mutants of cFBP1 (Livingston et al., 2010; Rojas-González et al., 2015). GOX1 is one of the peroxisomal flavin-dependent enzymes in the photorespiratory pathway that modulates reactive oxygen species-mediated signal transduction (Saji et al., 2017). Nitroblue tetrazolium (NBT) staining revealed that the HE and R lines had increased reactive oxygen tolerance after mimicking oxidative stress with different concentrations of sprayed paraquat (**Figure 2F**). In conclusion, EsNAC1 is involved in the regulation of pigment synthesis to improve plant resistance to oxidative stress.

### Redistribution of Carbohydrates Retarded the Vegetative Growth of the HE Lines

Because the heterologous expression of EsNAC1 retarded plant growth, the inference was that some growth regulators were negatively regulated by EsNAC1 in the HE and R lines. Compared with wild-type plants, c*FBP1* mutants and antisense-*cFBP1* plants accumulate less starch and have reduced contents of Ch a and b and carotenoids (Car) (Livingston et al., 2010; Rojas-González et al., 2015), whereas plants with higher relative expression of *cFBP1* accumulate more starch (Rojas-González et al., 2015), consistent with the performance of the HE lines (**Figures 5C,D**). *PKP1* encodes a chloroplast pyruvate kinase alpha subunit (Baud et al., 2007), and the enzyme catalyzes the conversion of phosphoenolpyruvate (PEP) to pyruvate with the concomitant formation of ATP. EsNAC1 upregulated this enzyme in *Arabidopsis* (**Table 1**). Pyruvate is the crucial precursor substance for the tricarboxylic acid (TCA) cycle in which ATP is synthesized. Heterologous expression of *EsNAC1* increased the accumulation of starch in *Arabidopsis*, which was accompanied by the increase of fructose 6-phosphate and downregulation of α-amylase-like 3 and β-amylase 4, 5, and 9 (**Supplementary Table S1**), this implied the starch accumulation in the HE lines by the modification of starch synthesis and decrease of starch catabolism in the light period. Additionally, sucrose must be hydrolyzed to guarantee a steady concentration of glucose and provide energy for abiotic stress resistance and plant growth. In conclusion, from the ratios of glucose, fructose and sucrose in Col-0 and the HE, M and R lines (**Figure 5E**), the heterologous expression of EsNAC1 caused the changes in sucrose, which were unfavorable for the growth of *Arabidopsis*. It could be concluded that EsNAC1 regulated the ratio of carbohydrates to be in favor of abiotic stress resistance and compromise growth for *Arabidopsis*.

Moreover, the *TRP2* gene (AT5G54810), which encodes a tryptophan biosynthesis enzyme (Zhao et al., 1998) in chloroplast and plasma membranes and has expression induced by oxidative, salt and drought stress treatments (Kilian et al., 2007), and the pyridoxal-5’-phosphate-dependent enzyme family protein gene (AT5G28237, encodes the beta chain of tryptophan synthase in mitochondria), were both upregulated in the HE lines (**Figure 4** and **Supplementary Table S1**). The *trp2* mutants showed tryptophan deficiency and smaller shoots due to retarded cell expansion (Jing et al., 2009). We also tested that ANTHRANILATE SYNTHASE ALPHA SUBUNIT 1 (not a direct downstream gene of EsNAC1 in *Arabidopsis*), which catalyzes the rate-limiting step of tryptophan synthesis (Jing et al., 2009) and was clearly downregulated in the HE lines (**Supplementary Table S1**). Trp is not only essential for protein synthesis but also a very important precursor for the synthesis of auxins, phytoalexins, glucosinolates and indole- and anthranilate-derived alkaloids, which play roles in regulating plant development, pathogen defense response and plant–insect interaction (Radwanski and Last, 1995; Woodward and Bartel, 2005). Thus, the heterologous expression of EsNAC1 produced complex effects on tryptophan synthesis.

### Differences in the Target Genes of EsNAC1 Caused Distinctions Between the HE and RD26OX Lines From *Arabidopsis*

EsNAC1 is the ortholog of RD26 in *Arabidopsis* but did not show identical target genes in *Arabidopsis*, and some of the regulated metabolic pathways were also different (**Figure 7**). Compared with the RD26OX line, the HE lines of EsNAC1 showed higher resistance to salt and oxidative stress and more obvious retardation in plant growth. A previous study in our lab identified the binding motif of EsNAC1 as CATG (Liu et al., 2017). Decades of classic studies show that RD26 recognizes and binds to regions containing a CACG consensus sequence (Fujita et al., 2004; Tran et al., 2004). The different expression patterns of the target genes of EsNAC1 and RD26 could be related to the distribution of CATG and CACG in the promoter regions. Additionally, the distance from the binding site to the TATA box may be an important factor determining whether a gene can be activated by these TFs. In the promoter regions of the candidate target genes of EsNAC1 in *Arabidopsis*, the binding motifs of EsNAC1 were distributed more widely than those of RD26, and distances from the TATA-box to CATG were much shorter than those to CACG. The differences in the target genes caused the differences between the HE lines and RD26OX lines, and the difference between the binding motifs of EsNAC1 and RD26 supported this hypothesis.

Brassinosteroids are crucial plant hormones regulating plant growth and abiotic and biotic stress responses (Clouse, 1996; Krishna, 2003). Ye et al. (2017) reported that genes in the BR signaling pathway were downregulated by RD26 to affect the vegetative growth of *Arabidopsis* by downregulating the expression level of BES1/BZR1 TFs. The expression pattern of the target genes of EsNAC1 in the BR signaling pathway was analyzed (**Figure 6**). The genes involved in the metabolism of nitrogen compounds in the HE lines showed a similar expression pattern to those of the partners in the BR signaling pathway, whereas over half of the genes that participated in primary metabolism showed an opposite tendency or varied independently in the HE and RD26OX lines. EsNAC1 might play an important role in the retardation of growth and increased abiotic stress resistance, similar to that of RD26 in the OX plants. The inference is that the heterologous expression of an orthologous TF in a related species might introduce its own specific characters by targeting different genes, which could result in advantageous effects in plant breeding.

## Author Contributions

CL, QS, and LZ performed most of the experiments and analyzed the data. ZL performed the data analysis and interpretation. ZP and JZ supervised the experiments and analyzed the data. CL and JZ completed the writing.

## Conflict of Interest Statement

The authors declare that the research was conducted in the absence of any commercial or financial relationships that could be construed as a potential conflict of interest.

## Footnotes

1 http://signal.salk.edu/tdnaprimers.2.html

2 https://figshare.com/projects/TsNAC1_ChIP-on-chip/25039

## Supplementary Material

FIGURE S1 Content of proline in transgenic lines, mutant lines and Col-0. The content of proline in HE and R lines was higher than that in Col-0 and the M lines under salt stress. All bars represent the mean ± SD, with three biological replicates in the experiment and each biological replicate with 20 plants; bars labeled with letters are significantly different at *P* < 0.05 by Duncan’s test.

FIGURE S2 Phenotypes of different lines in the TM-induced ER-stress. **(A)** Phenotypes of Col-0 and HE, M and R lines on 0.15 μg/mL TM 1/2 MS medium. **(B)** The phenotypic statistical parameters of different lines. Expanding cotyledons, and with normal cotyledons, indicated that seeds grew well on the 0.15 μg/mL TM 1/2 MS medium; germination represented plants that could germinate on the medium but with poorly developed cotyledons; non-germination represented plants that could not germinate on the medium. All the results were calculated from results of three independent experiments (36 seeds for each experiment). All bars represent the mean ± SD (*n* = 3).

FIGURE S3 Analysis results of Gene Ontology for the candidate target genes of EsNAC1 in *Arabidopsis.* Binding situation of EsNAC1 quantified by the percent of genes (*x*-axis) in different gene ontology terms (*y*-axis) (with the number of genes, *p*-value of enrichment) via the color of each square mapped to the peak scores.

FIGURE S4 Distribution of motifs recognized by EsNAC1 or RD26 in the promoter regions of some targeted genes. Blue square, CATG; Orange square, CACG; Green square, TATA-box predicted by the web service of PlantCARE.

FIGURE S5 Validity of EsNAC1 antibody. Western blot result of prokaryotic expressed EsNAC1-GST with the antibody of GST and EsNAC1.

TABLE S1 Some downstream-regulated genes by EsNAC1 in *Arabidopsis*.

TABLE S2 Candidate target genes of EsNAC1 in *Arabidopsis*.

TABLE S3 Primers used in this study.
