# Supplementary figures and images for: Heterologous Expression of the Transcription Factor EsNAC1 in Arabidopsis Enhances Abiotic Stress Resistance and Retards Growth by Regulating the Expression of Different Target Genes
Source: Front Plant Sci. 2018 Oct 15;9:1495. doi: 10.3389/fpls.2018.01495 (PMC6196249; doi:10.3389/fpls.2018.01495)

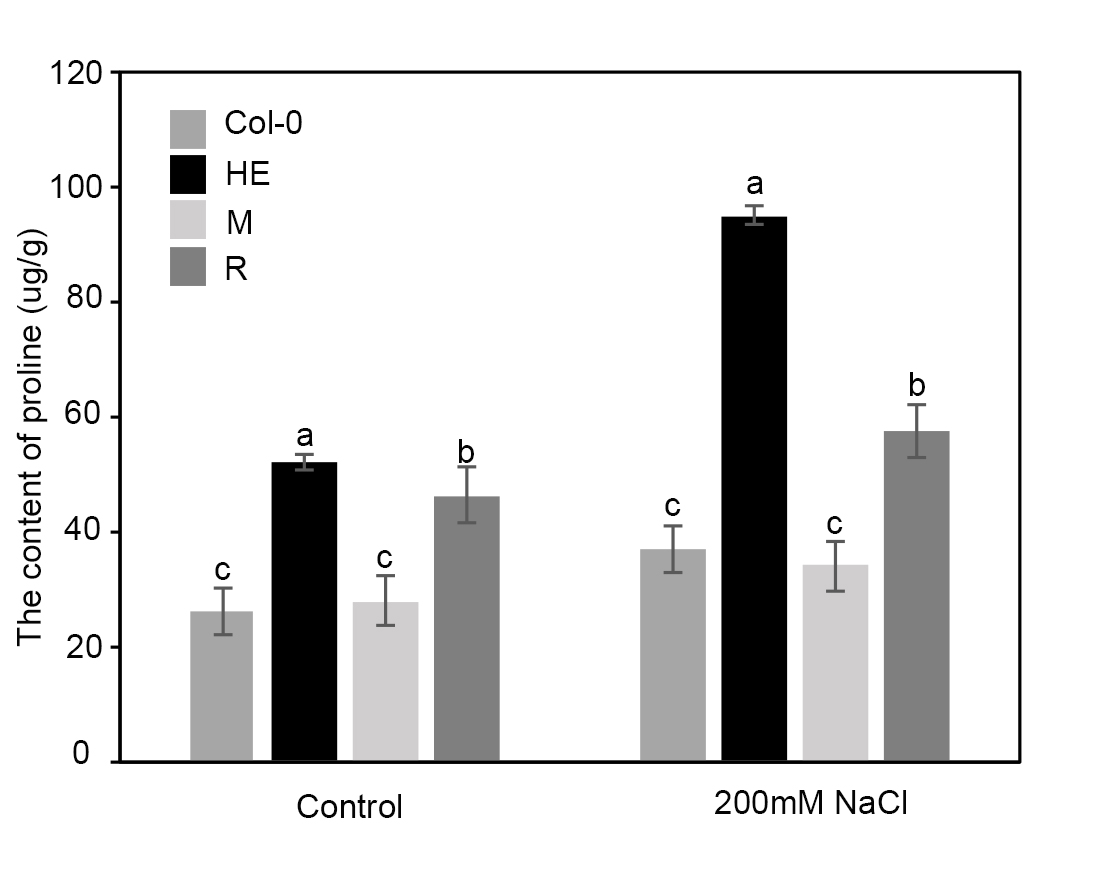

Supplement: FIGURE S1 — Content of proline in transgenic lines, mutant lines and Col-0. The content of proline in HE and R lines was higher than that in Col-0 and the M lines under salt stress. All bars represent the mean ± SD, with three biological replicates in the experiment and each biological replicate with 20 plants; bars labeled with letters are significantly different at P < 0.05 by Duncan’s test. [file Image_1.JPEG]

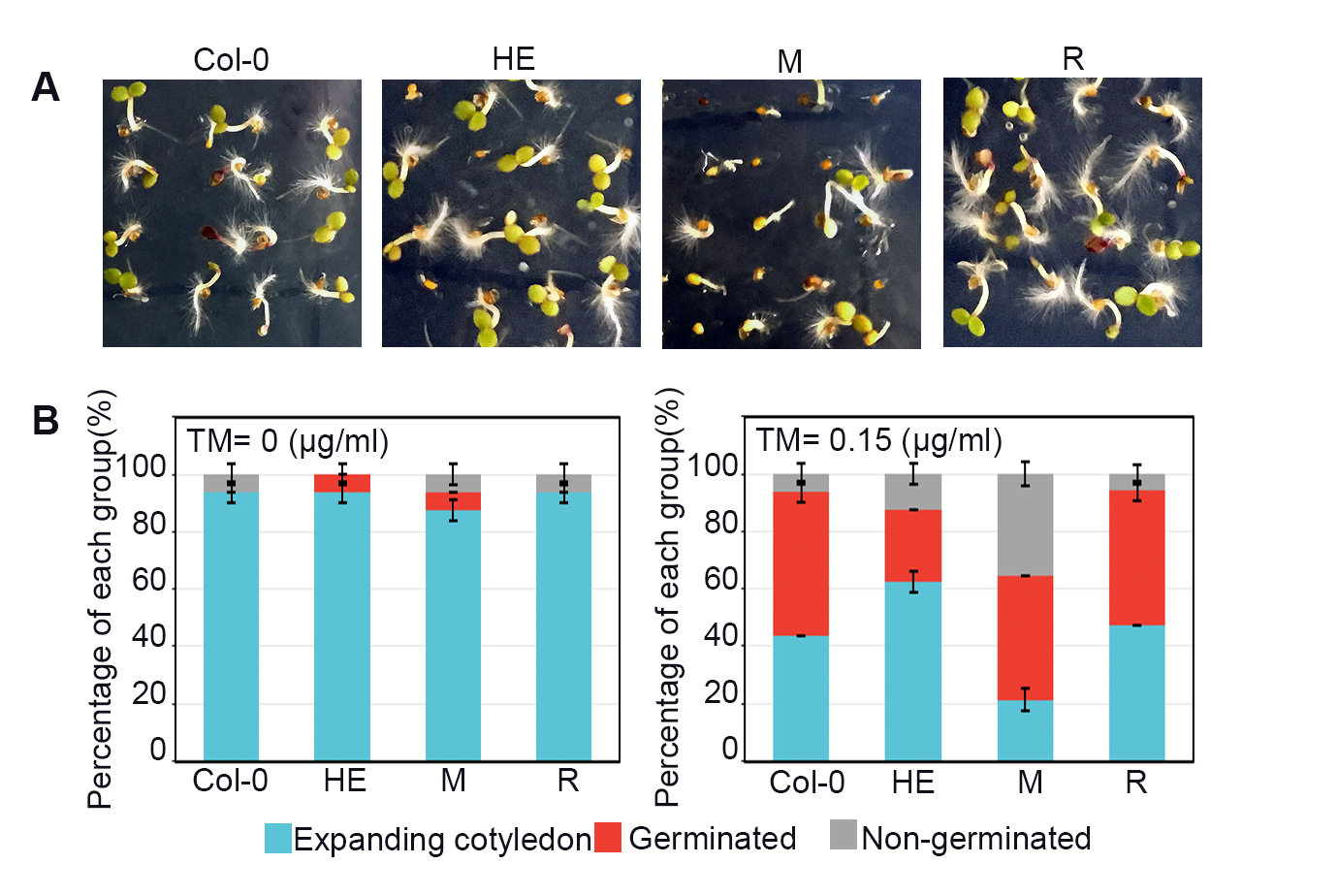

Supplement: FIGURE S2 — Phenotypes of different lines in the TM-induced ER-stress. (A) Phenotypes of Col-0 and HE, M and R lines on 0.15 μg/mL TM 1/2 MS medium. (B) The phenotypic statistical parameters of different lines. Expanding cotyledons, and with normal cotyledons, indicated that seeds grew well on the 0.15 μg/mL TM 1/2 MS medium; germination represented plants that could germinate on the medium but with poorly developed cotyledons; non-germination represented plants that could not germinate on the medium. All the results were calculated from results of three independent experiments (36 seeds for each experiment). All bars represent the mean ± SD (n = 3). [file Image_2.JPEG]

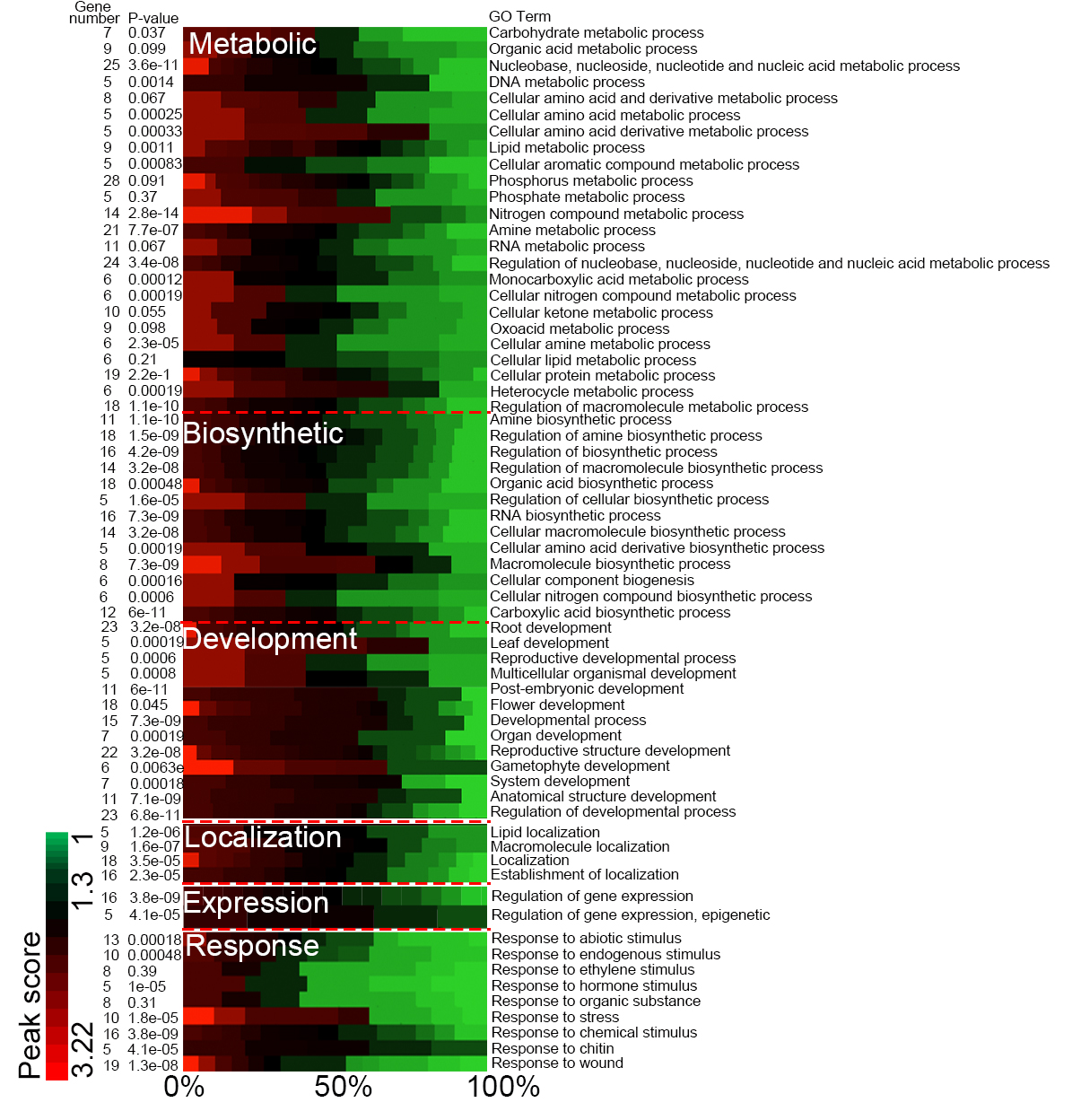

Supplement: FIGURE S3 — Analysis results of Gene Ontology for the candidate target genes of EsNAC1 in Arabidopsis. Binding situation of EsNAC1 quantified by the percent of genes (x-axis) in different gene ontology terms (y-axis) (with the number of genes, p-value of enrichment) via the color of each square mapped to the peak scores. [file Image_3.JPEG]

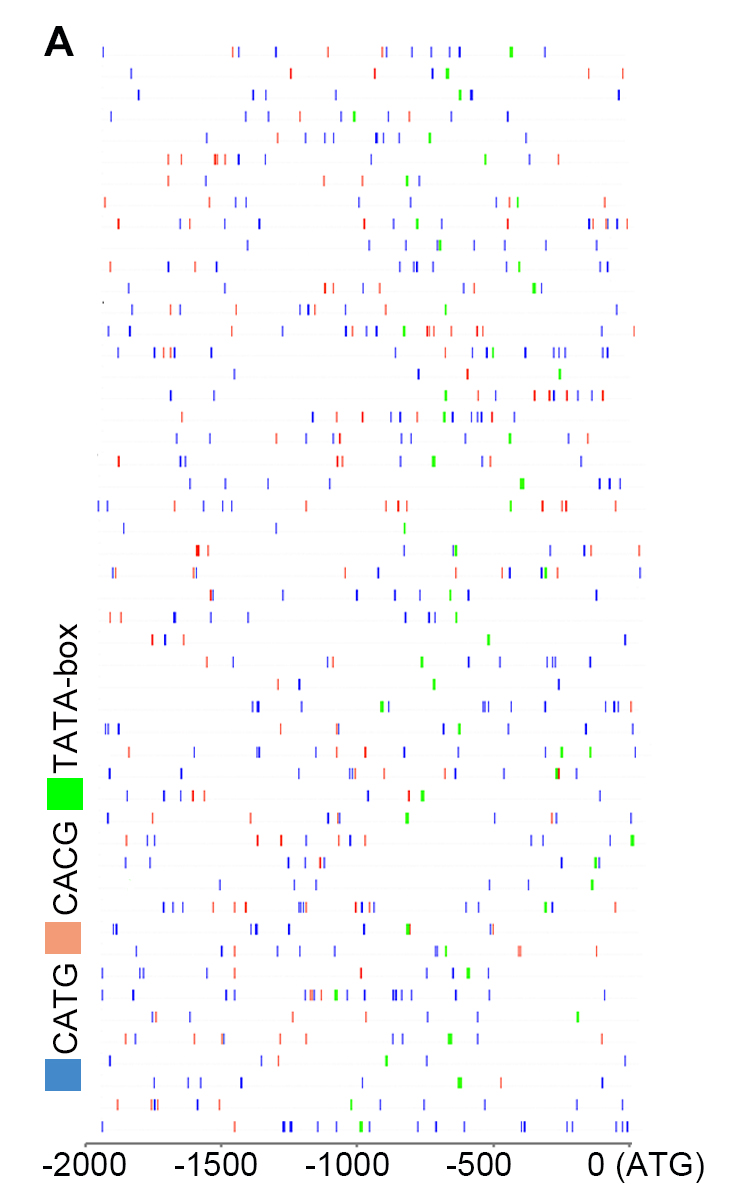

Supplement: FIGURE S4 — Distribution of motifs recognized by EsNAC1 or RD26 in the promoter regions of some targeted genes. Blue square, CATG; Orange square, CACG; Green square, TATA-box predicted by the web service of PlantCARE. [file Image_4.JPEG]

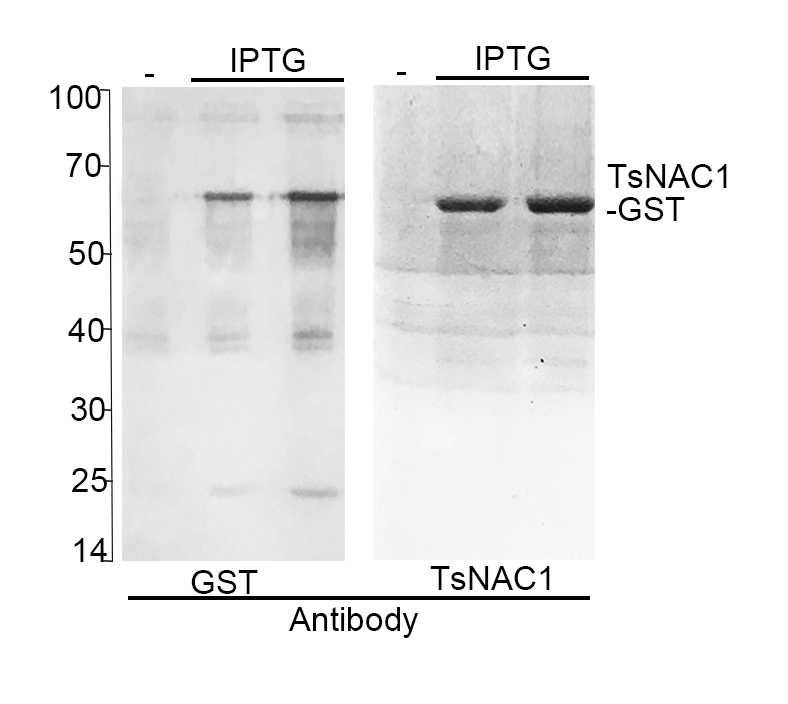

Supplement: FIGURE S5 — Validity of EsNAC1 antibody. Western blot result of prokaryotic expressed EsNAC1-GST with the antibody of GST and EsNAC1. [file Image_5.JPEG]
