# Supplementary material for: Heterologous Expression of the Transcription Factor EsNAC1 in Arabidopsis Enhances Abiotic Stress Resistance and Retards Growth by Regulating the Expression of Different Target Genes
Source: Front Plant Sci. 2018 Oct 15;9:1495. doi: 10.3389/fpls.2018.01495 (PMC6196249; doi:10.3389/fpls.2018.01495)
Supplement: TABLE S1 — Some downstream-regulated genes by EsNAC1 in Arabidopsis. [file Table_1.DOCX]

| AGI^a^ | Full Name | REL(+)^b^ |
| --- | --- | --- |
| AT5G39550 | Zinc finger (C3HC4-type RING finger) family protein (VIM3) | 1.95 |
| AT1G57820 | Zinc finger (C3HC4-type RING finger) family protein (VIM1) | 1.88 |
| AT1G74430 | Myb domain protein 95 (MYB95) | 2.53 |
| AT5G09690 | Arabidopsis thaliana magnesium transporter 7 (MRS2-7) | 1.61 |
| AT4G34720 | VACUOLAR H+-PUMPING ATPASE C1 (VHA-C1) | 1.57 |
| AT5G28237 | Pyridoxal-5-phosphate-dependent enzyme family protein | 3 |
| AT2G45880 | Beta-amylase 4 | 0.25 |
| AT2G32290 | Beta-amylase 5 | 0.41 |
| AT1G69830 | Alpha-amylase-like 3 | 0.56 |
| AT4G00490 | Beta-amylase 9 | 0.63 |
| AT5G05730 | Anthranilate synthase alpha subunit 1 | 0.68 |

Table S1. Some downstream genes regulated by TsNAC1 in *Arabidopsis*

AGI^a^, Arabidopsis Genome Initiative.

REL(+)^b^, Log2(relative expression level in HE lines/Col-0).
