# Supplementary material for: Heterologous Expression of the Transcription Factor EsNAC1 in Arabidopsis Enhances Abiotic Stress Resistance and Retards Growth by Regulating the Expression of Different Target Genes
Source: Front Plant Sci. 2018 Oct 15;9:1495. doi: 10.3389/fpls.2018.01495 (PMC6196249; doi:10.3389/fpls.2018.01495)
Supplement: TABLE S3 — Primers used in this study. [file Table_3.DOCX]

| Table S3. Primers used in this study. | | | |
| --- | --- | --- | --- |
| Locus name | Gene name | Forward | Reverse |
| For gene clone | | | |
| TP7G25350 | TsNAC1 | GGAAGAATCCAAAGTCAGAGGA | ACATCAACTGCCACTCTACCCT |
| For T-DNA insert mutants identify | | | |
| SALK_072276 | RD26 | TTAATGCATGTGTGGGTGATG | CCCGGAGATAAAAGACCAGTC |
| SALK_083756 | RD26 | GACTGGTCTTTTATCTCCGGG | ACAACACATCGATAAGGTCGG |
| For one-hybrid yeast system assay | | | |
| AT3G49530 | NAC062 | TCCTTGGGTCTACTCGCACA | AGAAGGCAGATGTGAGCTTTG |
| AT5G11590 | TINY2 | ACCCCCTTTAGTACACCTTTTCA | AAGGAAATGGACCCAGTGGC |
| AT5G47390 | MYBH | GACTTTTGGAGTTAGGGGGTGT | TATTTGGTGTATCGTTGGTGGA |
| AT3G55120 | CFI | AACGTTCGTTCCCTCGAGTC | TGGTTGAAGCTTTGAGCCCT |
| AT2G39800 | P5CS1 | ATGAATGAAGGCAGCGGACA | GCGAACGTATCTCAACGAACC |
| AT5G54810 | TRP2 | GTGTTTTCTGCATTCTGCGG | AGCAGACACATGTAAGCAGACC |
| At1g56600 | GOLS2 | AAATGTAGAATGGGGACTTTTTCAT | TGGGTGATGCTTAATGGTGCT |
| AT3G54050 | CFBP1 | TTGTCGAGTTGCTGCTATGGT | ATGACACGTGGCTTTGCTCT |
| AT3G22960 | PKP1 | TTTCTCGGCCCACAGAAGTA | TGCCGTCGAGGCTGTATGTA |
| AT3G14420 | GOX1 | AGAAACAACACGGCACCAAA | GAGGAACATGTCCATGAACGC |
| For ChIP-qPCR assay | | | |
| AT3G49530 | NAC062 | TTTAGCCGGTCCTAGCTCCA | CCCTTGAGCGACTTTGGGAT |
| AT5G11590 | TINY2 | TGCCTTCACATGATGAGCATA | AGAAGGGCAGTGTTGAAAAGAGA |
| AT5G47390 | MYBH | GACTTTTGGAGTTAGGGGGTGT | AATAGCCCTTCACGCGTTCC |
| AT3G55120 | CFI | AACGTTCGTTCCCTCGAGTC | AGTTCTGATGCCGGGAAGTG |
| AT2G39800 | P5CS1 | ATGAATGAAGGCAGCGGACA | AACCGTCAACGGATCAGACC |
| AT5G54810 | TRP2 | GTATCCCAATTCCCAACTTGTGT | AGCAGACACATGTAAGCAGACC |
| At1g56600 | GOLS2 | GCCGTAAATAGTCGGTCGGT | ATAATCCAGGTGCTGCCACG |
| AT3G54050 | CFBP1 | GCAGAGCACATCAGCCAGAA | GGCTTTGCTCTTACGGACCT |
| AT3G22960 | PKP1 | GCTAGCAATGCGGAATCGAC | CGTTCTCGCCAAATCGAATGA |
| AT3G14420 | GOX1 | ACCTCCAGCTTCTCATCCAT | AGCAAAAAGGCCTGAGTTAGC |
| [AT5G62690](http://www.arabidopsis.org/servlets/TairObject?id=134382&type=locus) | TUB2 | ACACAGAGAGGAGTGAGCAA | CGGTTGGATGAGTGAACGGA |
| For RT-PCR assay | | | |
| AT3G49530 | NAC062 | ATTGGGTGACAAATGTGGAG | GTCAATGTAGCAGCGTGTTT |
| AT5G11590 | TINY2 | TGCTGGTCGTATTGCTGGAT | CTGCCTCCGTCAACCTCTG |
| AT5G47390 | MYBH | TCATCAGACACTTGCTCCTAGC | TTTCGAGTGCACAGCAGTTG |
| AT3G55120 | CFI | ACGCCGTTCCTTCTCTATCTG | GAGGATCGATGAACCGGGAG |
| AT2G39800 | P5CS1 | GGTCTTTACACAGGCCCTCC | AACACGGCCGATTGGATCTT |
| AT5G54810 | TRP2 | CTCGCTCCAAGTCATCATCCT | TCAGATAGAGCGTGCATAAGG |
| AT1G56600 | GOLS2 | CGCCACAGTACAAGATCGGT | GGCTGTCCCCAATGACAACA |
| AT3G54050 | cFBP1 | GAGCCGAGCCAGGTAAAGT | TTGAAGCCGTGTATGAGCC |
| AT3G22960 | PKP1 | AACCCGTCATTGTCGCTTCA | GTGATGCCATGTGTCCGCT |
| AT3G14420 | GOX1 | GCTCCAACTGCCATGCAAAA | AAGCCAGAGTCATTGGCCTC |
| AT4G34640 | SQS1 | \| CCCGCAACCCTCTGATAACT \| TTGAAACGCCGCCCTATT \| \| --- \| --- \| | TTGAAACGCCGCCCTATT |
| AT3G02630 |  | ACCAGGTTACCAACAGTGCA | GTGATCGAACAAACGGCTC |
| AT3G24800 | Proteolysis 1 | AAGAAGCCACCTCGTCTCAC | GCCAGCATAAACCACAGTT |
| AT3G49370 | CDPK | ACACCGCCTTTCAATAATCT | AACTGTCACCGGAATCATCT |
| AT3G32029 |  | AGGAGACGGGTCATCGAGTT | CCTCTTCTTCACCGAGCAAC |
| AT5G63690 |  | TTGTACCGTCCGTCAAGTC | CAGTCCGTATAAAGCCCTAA |
| AT4G34200 | EDA9 | TGCTTCTGGCAATCCACTTAC | TTACCAACGGAACAACAACG |
| AT3G09070 | DUF740 | TGGTAGGAATGTTGGGTTAAG | AAACGGTCACAGAAGTAGACAT |
| AT5G39550 | VIM3 | CCTAAACGATACACTCCACCTAA | GTCAAGTCCCGCTCAAGAAAC |
| AT1G57820 | VIM1 | CTTGTAGCTGCGATTCGTGC | CCTTGGTTCCTGACAGGGTC |
| AT1G74430 | MYB95 | TCTTGGAAACAGGTGGGCAG | ACCGTCTCTGTATGGGTCGA |
| AT5G09690 | MRS2-7 | GTGGATTCATCGGCGGTAGT | AAACGGGTCATGGCACTCTT |
| AT4G34720 | VHA-C1 | CTCCCTTCTTCGGCTTCCTC | GCCAGCTCGTGAGGAAAGAA |
| AT5G28237 |  | CCCCTTCCATCAGCCATCT | AAGGCGTCACAGAGCGAGAT |
| AT2G45880 | BMY4 | CAGGGGACAAAGCCTACTGG | ATCATACGGTGATGGAGCGG |
| AT2G32290 | BMY5 | GTGCGATTGCGAAAATAATGC | AACGGAAGCAGCTCGTGATTA |
| AT1G69830 | AMY3 | CTGAAGCTGCCCAAGTGAGT | CCTTCCTCGGAAAAAGCTGG |
| AT4G00490 | BMY9 | TTGGAGCTCCGACGAGAGTA | CGAGAACCAAAGGCCTAGCA |
| AT5G05730 | ASA1 | AGGCCCCTGAGGATGATAGG | TTCTCTCCATCACCAACCTGC |
